# Density-Based Detection Rapid Exploration Random Tree for Multirobot Formation Cooperative Path Planning

**DOI:** 10.3390/s25072201

**Published:** 2025-03-31

**Authors:** Yuzhuo Shi, Yang Yang, Jinjun Liu, Kun Hao, Jiale Zhao, Haoyi Chai

**Affiliations:** 1 College of Information Technology, Tianjin College of Commerce, Tianjin 300350, China; 2 School of Computer and Information Engineering, Tianjin Chengjian University, Tianjin 300384, China; 3 School of Control and Mechanical Engineering, Tianjin Chengjian University, Tianjin 300384, China; 4 School of Information and Communication Engineering, Hainan University, Haikou 570228, China

**Keywords:** multirobot formation, density testing, RRT algorithm, artificial potential field method, consistency control

## Abstract

This paper proposes a multirobot formation path planning method based on the leader–follower formation control method to ensure smooth operation in the multirobot formation control area. First, on the basis of the rapidly exploring random tree (RRT), a density detection rapidly exploring random tree (DDRRT) algorithm is designed to avoid repeated exploration of by the RRT, to quickly generate a global path from the starting point to the destination for the leader robot, and to propose a rope shrinkage path optimization mechanism for path optimization. Second, the repulsion field function in the artificial potential field (APF) is optimized for local collaborative obstacle avoidance to enable multiple robots, and a rotational potential field is introduced to solve the problems of unreachable targets and local oscillations. Finally, a control law based on consistency control is used to control the followers and introduce a formation change mechanism based on polar coordinate transformation to enhance the formation control capability. The simulation results show that the proposed strategy can provide high-quality paths for robot formations in multiple obstacle areas and guide robot formations to avoid various local obstacles quickly through formation transformation.

## 1. Introduction

With the rapid development of science and technology, multirobot systems have become the focus of advanced research and applications in the field of robotics. The application fields of multirobot systems continue to expand from industrial manufacturing to intelligent transportation, and then to service robots [1]. For example, in industrial manufacturing, a specific task requires multiple robots to work together [2]. In the field of intelligent transportation, multirobot systems can be used in autonomous driving fleets to cooperatively complete vehicle navigation and obstacle avoidance tasks, improving safety and efficiency in road traffic [3]. In the field of service robots, for example, medical robot teams can cooperate to complete surgical tasks, which improves the accuracy and safety of surgery [4]. In these application scenarios, the collaboration and cooperation of multirobot systems are critical, and path planning and formation control are key technologies for ensuring their efficient operation. As a key technology in multirobot systems, path planning can be further divided into global path planning and local path planning.

The sampling-based path planning algorithm in global path planning [5] uses a sampler to sample the unknown space and obtain space information. With increases in the number of sampling points, the unknown space can be described with higher resolution so that feasible paths can quickly be found. The rapid exploration random tree (RRT) algorithm is widely used. The collision checking module can maneuver in complex spaces and is thus suitable for solving high-dimensional or multiconstraint planning problems [6]. However, due to the random sampling nature of the RRT algorithm, the paths it generates often suffer from poor quality, such as excessive length, too many turning points, and other inefficiencies. Additionally, RRT cannot be guaranteed to find the globally optimal path, especially in complex environments, where its search efficiency is low, and it is prone to falling into local optima. These limitations restrict the application of RRT in practical multi-robot collaborative tasks, necessitating an improved method that can optimize path quality and enhance search efficiency. To address this problem, many scholars have proposed improved methods. To solve the problem of low-quality generated paths, Karaman et al. [7] proposed the RRT* algorithm, which introduces the ChooseParent and Rewire procedures when new nodes are added to the tree, making the algorithm asymptotically optimal. Bao et al. [8] proposed a method to optimize the initial path generated by RRT; that is, the tree is merged on the basis of the initial path to form a closed-loop path, and then optimization is carried out to obtain a relatively optimal path. Chen et al. [9] proposed a bidirectional pruning optimization strategy to prune redundant nodes from the starting point and end point of the path and select the shortest optimized path to effectively improve the quality of the path. However, the RRT* algorithm needs infinite iterations to find the optimal path. Although the pruning optimization improves the quality of the path, there is a gap between it and the optimal path.

As a supplement to global path planning, local path planning focuses on how to avoid obstacles and adjust the path in real time during the robot’s movement. As one of the classical algorithms of local path planning, the artificial potential field method is simple and efficient and is widely used today. However, the original algorithm has problems, such as an unreachable target and local optimality [10]. In this context, many scholars have made various improvements to the algorithm. Fan et al. [11] adopted the regular hexagon guidance method to guide the robot along one side of a virtual regular hexagon centered on an obstacle to escape the local minimum. Jia et al. [12] proposed a new repulsion force algorithm that adds Gaussian functions related to the position of the robot and the target point on the basis of the traditional repulsion force to solve the unreachable problem of the target point near the obstacle. Sun et al. [13] used the dynamic window method and evaluated the simulation trajectory to predict the local minimum region to prevent the robot from falling into the local minimum point in advance. However, the above improvements do not consider the problem of cooperative obstacle avoidance among multiple robots.

The consistency and stability of multirobot formations are also important problems in multirobot systems. Ge et al. [14] applied the consistency control law to the formation control problem of multiagent systems and proved that several traditional formation strategies, such as the leader–follower strategy, can be realized by using the consistency theory. Lin et al. [15], based on the symbolic Laplacian matrix of the multirobot affine formation control method, analyzed the conditions of the affine transformation realization of multirobot systems, providing a new method for multirobot formation control. Zhao et al. [16] studied the affine formation control method corresponding to the undirected graph stress matrix to provide sufficient and necessary conditions for robot formation to realize affine positioning. Different formation controllers based on affine transformation are designed under different conditions, and the stability and global convergence of the proposed control methods are proven. However, these strategies have their limitations and disadvantages, which limit their wide application in complex scenarios.

In summary, although many studies have focused on path planning and the formation control of multiple robots, there is a lack of research on their ability to maintain formation stability, and follower loss easily occurs in multiple obstacle regions. Therefore, in this paper, the leader–follower method is used as the premise for formation control, and a global path planning algorithm based on the RRT algorithm, namely, the density detection rapidly exploring random tree (DDRRT) algorithm, is proposed. In this algorithm, a density detection mechanism is added to the RRT algorithm extension, and tree nodes whose density values exceed the threshold are deactivated to avoid invalid expansion and shorten the pathfinding time. Additionally, a rope contraction path optimization method is proposed. The randomly generated path is treated as a rope, with its starting point fixed while the endpoint is adjusted to shorten the overall path, resulting in a path close to the optimal solution and providing a high-quality path for the robot.

Furthermore, the potential field function of the artificial potential field method is improved. The repulsive force potential field is adjusted to avoid force balance and prevent the robot from falling into local optima. A rotating potential field is added around obstacles to increase the robot’s obstacle avoidance speed and eliminate local oscillation movements. Finally, the consistency algorithm is used to realize multirobot cooperative formation operations, and a formation transformation mechanism based on polar coordinates is proposed to enable formation steering and obstacle avoidance, thereby improving the stability of multirobot formation operations.

## 2. Global Path Planning via the DDRRT Algorithm

### 2.1. DDRRT Algorithm

#### 2.1.1. Algorithm Framework

The RRT explores space by maintaining a tree, as *T* = (*V*,*E*), where *V* is the vertex set of the tree and *E* is the local path set between vertices. The DDRRT algorithm adds a density detection mechanism and a target probability orientation mechanism on the basis of the RRT algorithm framework. Algorithm 1 gives the pseudocode of DDRRT. The algorithm sets the tree root node to *x_start_* and performs iterative expansion. Then, the sampler is modified so that the sampling point *x_rand_* is sampled to the target range with a certain probability in each iteration, increasing the growth guidance of the tree (line 3 of Algorithm 1). The vertex *x_nearest_* is then found to be closest to the sample *x_rand_* from *V* (Line 4 of Algorithm 1). Then, starting from *x_nearest_*, it is extended by a step *d_stepsize_* towards *x_rand_* direction, and then obtains the node for expansion *x_new_* (lines 4–5 of Algorithm 1). A density detection function *CheckDensity* is added to detect whether the tree node density is reasonable. When obtaining *x_nearest_*, only nodes with reasonable densities are compared, and only *x_new_* with reasonable densities are expanded (Line 6 of Algorithm 1). Finally, we enter the collision detection stage. If the local path *E_local_* from *x_nearest_* to *x_new_* is collision-free, *x_new_* and *E_local_* are added to the tree (lines 7–11 of Algorithm 1). When a feasible path is obtained, or the number of iterations exceeds the maximum number of iterations *N*, the algorithm stops.
**Algorithm 1** DDRRT(x_start_, x_goal_, d_stepsize_, d_obs_, N, Map)1: V = {x_start_}; E = [ ];  2: for i = 1 to N do 3:    x_rand_ = GetSample(i); 4:    x_nearest_ = GetNearest(V, x_rand_); 5:    x_new_ = GetSteer(x_near_, x_nearest_, d_stepsize_); 6:   if CheckDensity(V, x_new_, d_check_,Map) then continue end if 7:   if CollisionFree(x_new_, x_nearest_, Map) then 8:     V = V {x_new_}; 9:     E = E {(x_nearest_, x_new_)}; 10:      if x_new_ X_goal_ then 11:      T = (V, E); 12:      return X = GetPath(T); 13:      return 14:   end if 15: end for

#### 2.1.2. Density Detection Extension Mechanism

The traditional RRT algorithm randomly samples the configuration space to guide tree expansion exploration. When the RRT algorithm extends a new vertex, RRT calculates the distance *d* between the new vertex and the target point. If *d* is less than the threshold *r*, the algorithm finds the target point until the search ends; otherwise, the search continues. Therefore, every time a new node is expanded, the RRT algorithm explores the region centered on the node with the threshold value *r* as the radius, and we define this region as the exploration region. (In this paper, the detection threshold is set as equal to the expansion step). The RRT algorithm finally explores the entire space through continuous sampling and expansion. However, each exploration will inevitably involve secondary exploration of some areas, and it is also possible that some already explored areas will be explored repeatedly more than three times. According to the new expansion vertex, the exploration efficiency is set through the exploration area of the unknown region and the area and number of repeated explorations of the known region. The larger the area of exploration of the unknown region is, the higher the efficiency. The larger the area of exploration of the known region is, the greater the number of exploration iterations, and the lower the exploration efficiency.

**Figure 1:**
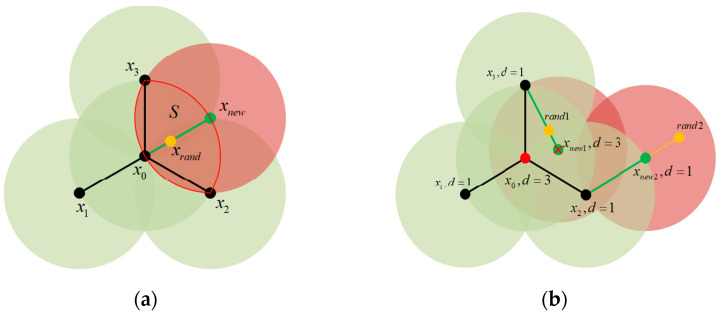
Schematic diagram of density detection. (**a**) Shows the repeated exploration of the known area. (**b**) Illustrates the expansion after adding density detection.

Figure 1a shows repeated explorations of a known region. When a new vertex explores a region more than three times, it is named an *S* region. Figure 1a shows the traditional RRT extension process. In the figure, the region wrapped by the red line is the *S* region. Vertices *x*_0_, *x*_1_, and *x*_2_ have completed two repeated explorations of the *S* region. Then, vertex x_1_ expands to a new point *x_new_* in the *x_rand_* point direction. The new vertex will conduct three repeated explorations of the *x_new_* region. Exploring the same region multiple times is obviously meaningless.

To address these problems and minimize the number of repeated explorations in the exploration region, a density detection expansion mechanism is proposed. To increase the density perception ability of each vertex, the density value of the vertex is set to the number of other vertices in the vertex exploration region. When the density value is 3, such as point *x*_0_ in Figure 1a, its exploration region is surrounded by the exploration region of surrounding points *x*_1_, *x*_2_, and *x*_3_. When *x*_0_ is expanded in any direction, the exploration region of *x*_0_ will be explored more than three times. To reduce multiple repeated samples of the exploration region, the density threshold *v* is set to 3. When the density value of a vertex is greater than or equal to *v*, the vertex will be inactivated, and the inactivated vertex will not be selected as *x_near_* for extended exploration. In addition, density detection is also performed for the expansion of new nodes. If the density value of the new node is not less than *v*, the expansion fails, and resampling is performed. As shown in Figure 1b, the red vertex *x*_0_ in the figure is an inactive vertex. Because its density value, *d = 3*, is not less than the threshold value *v*, inactive vertices will no longer participate in the expansion process of the random tree. Therefore, although the sampling point *x_rand_*_1_ is closest to the vertex *x*_0_, because it is an inactive node, it will not be selected as the *x_nearest_* vertex. Later, *x*_3_ is selected as the *x_nearest_* point to expand the *x_new_*_1_ vertex. However, the density value of this new vertex is *d > v*, so the expansion fails and resampling expansion is performed. The resampling point is the *rand2* vertex, and *x*_2_ is selected as the *nearest* to expand the new *x_new_*_2_ vertex. The density value of the *x*_2_ vertex is 1, and the density value of *x_new_*_2_ is 1 and less than or equal to *v*. The results show that the new vertex *x_new_*_2_ meets the density constraint. More than half of its explored region is unexplored, and less than 10% of the region is explored three times, which makes vertex exploration more efficient. When the exploration efficiency of each vertex is improved, the exploration efficiency of the whole algorithm will also be effectively improved, which is reflected in the algorithm execution time, the number of samples, and the total number of RRT vertices. Algorithm 2 gives a method to detect the density of the *x_new_* vertex.
**Algorithm 2** CheckDensity(V,x_new_, d_check_,v)1: n = *GetNearNodeNum*(*V*,*x_new_*, *d_check_*) 2: **if**
*n > v*
**do** 3:    **return**
*True*; 4: **else** 5:    **return**
*False*; 6: **end if**

### 2.2. Rope Contraction Path Optimization Strategy

**Figure 2:**
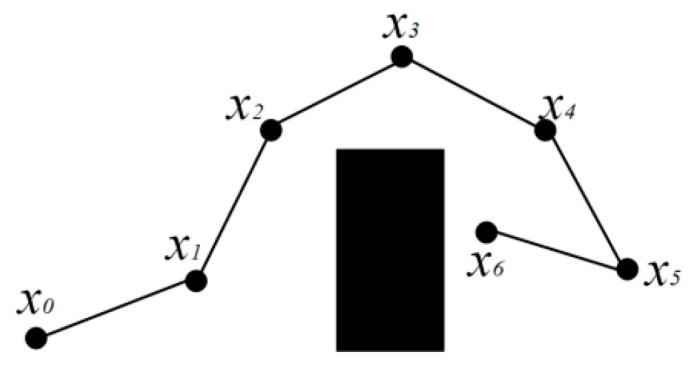
Initial RRT path.

Because the path finding process of the sampling algorithm is random, many redundant nodes are generated, so the quality of the generated path is not satisfactory. In this paper, a path optimization strategy simulating rope contraction is proposed to improve path quality. As shown in Figure 2, moving from *x*_0_ to *x*_6_ requires avoiding an obstacle, and the black path *x*_0_-*x*_1_-*x*_2_-*x*_3_-*x*_4_-*x*_5_-*x*_6_ in the figure is the planned original path. This path has many redundant points and poor path quality, which makes it difficult to serve as a global path guide leader. Therefore, a rope contraction path optimization strategy is proposed. The path is simulated as a rope, and the path points other than the starting point move toward the next path point successively, with the moving distance *d_step_* and *d_step_* < *d_stepsize_*. The strategy is divided into two processes: collision-free contraction and collision regression.

**Figure 3:**
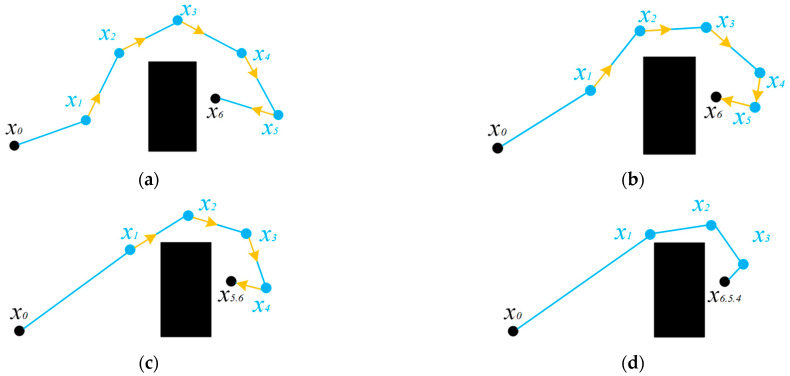
Path point collision-free contraction process. (**a**) First contraction, (**b**) Second contraction, (**c**) Third contraction, (**d**) Fourth contraction.

The collision-free contraction process is shown in Figure 3. Start point *x*_0_ and end point *x*_6_ do not participate in contraction movement. Therefore, the start point and end point can be defined as inactivated path points, denoted by black dots. The remaining points can be defined as movable path points and are represented by blue dots. In each round of contraction, each movable path point moves a distance in the direction of the previous path point in turn. In Figure 3a, the yellow vectors represent the movement direction and movement length of the path points. After each path point moves, it is necessary to detect whether the path collides. If there is no collision, the next path point continues to move. When all path points have completed movement, a round of contraction is completed. The second and third rounds of contraction then began.

**Figure 4:**
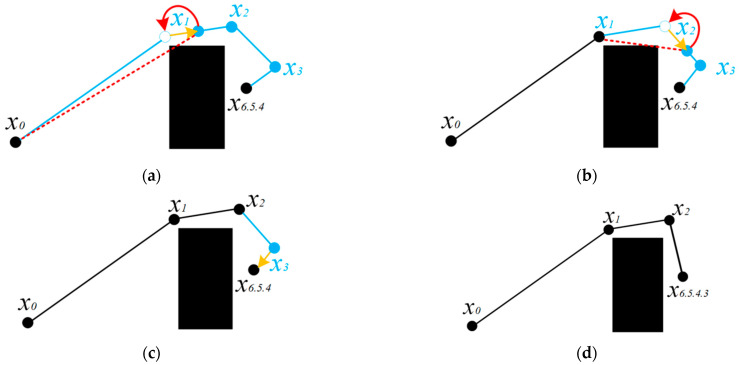
Path point collision–regression process. (**a**) The contraction process of node *x*_1_ encountering an obstacle. (**b**) The contraction process of node *x*_2_ encountering an obstacle. (**c**) The contraction process of node *x*_3_. (**d**) The final optimized path.

When a movable path point meets an inactive path point, the movable path point becomes an inactive path point, and the two points are merged into one path point. In Figure 3b, the *x*_5_ path point encounters the inactive path point *x*_6_ after the next move. After moving, as shown in Figure 3b, *x*_5_ becomes an inactive path point and is merged with *x*_6_ into one path point. When a path point moves and causes the path to collide with an obstacle, it enters the path point collision–regression process. We then rewind the current path point to the position before the move, and mark the path point as an inactivated path point. The subsequent path points continue to move, as shown in Figure 4. In Figure 4a, the line between path point *x*_1_ and path point *x*_0_ collides with the obstacle after path point *x*_1_ moves. At this time, *x*_1_ returns to the position before the move, and the point is deactivated. The *x*_2_ path point continues to move and detects whether a collision has occurred, as shown in Figure 4b. After detection, a collision will occur. The *x*_2_ point then returns to the position before the move and is deactivated, as shown in Figure 4c. The subsequent path points continue to shrink and move until all path points become inactive points. At this time, the new path obtained is the path optimized by rope contraction, as shown in Figure 4d. Compared with the original path in Figure 2, the new path has a shorter path length and no redundant points. It has more advantages as a global path guide leader.

Algorithm 3 presents the pseudocode of the rope contraction path optimization strategy. First, the path points are initialized to two states: inactive, “Dead”, and movable, “Alive”. The start point and end point are “Dead”, and the remaining path points are “Alive” (Line 1 of Algorithm 3). The circular movement is entered, and each cycle is a round of movement. In each round of movement, the movable points are moved toward the next path point in turn (Lines 3–4 of Algorithm 3). Whether a collision occurs during the movement is determined (Line 5 of Algorithm 3). If there is no collision, the algorithm continues to determine whether the path point will encounter the next path point (Line 6 of Algorithm 3). If there is an encounter, the state and position of the path point will be merged (Line 6 of Algorithm 3). If a collision occurs, the current path point returns to the position before the move, and the state is changed to “Dead” (line 2 of Algorithm 3). When the state of all path points is “Dead”, the contraction ends and exits the loop (Line 2 of Algorithm 3). The new path obtained at this time is the path optimized by rope search.
**Algorithm 3** PathOpt(*X*, *n*, *Map*)1: *X*.state = IntialState(*X*); 2: while AllStateDead(*X.state*) do 3:   for *i* = 1 to *n* − 1 do 4:      Move(X(i).pos, X(i + 1).pos); 5:      if CollisionFree(*X(i), X(i − 1), Map*) then 6:      if MeetNext(*X*(*i*), *X*(*i* + 1)) then 7:       *X*(*i*).*pos* = *X*(*i* + 1).*pos*; 8:       *X*(*i*).*state* = *X*(*i* + 1).*state*; 9:      end if 10:    else 11:       MoveBack(*X*(*i*).*pos*); 12:       StateDead(*X*(*i*).*state*); 13:    end if 14:    end for 15: end while 16: return *X*;

## 3. Obstacle Avoidance Strategy Based on an Improved Artificial Potential Field Method

### 3.1. Improvement of the Potential Field Function

In traditional artificial potential fields, there are problems in which the target is unreachable and easily falls into local oscillation. When an obstacle is located on the line between the target point and the mobile robot, a situation where the attraction and repulsion are equal and in opposite directions may occur, causing the robot to stop running and be unable to reach the target. In a channel environment, if there are obstacles on both the left and right sides to generate repulsion, the robot may generate oscillatory motion, resulting in reduced obstacle avoidance efficiency. To solve the above problems, this paper introduces a new repulsion function to address oscillatory motion and introduces a rotating force field to solve the problem of a target being unreachable.

#### 3.1.1. Improvement of the Repulsion Field

**Figure 5:**
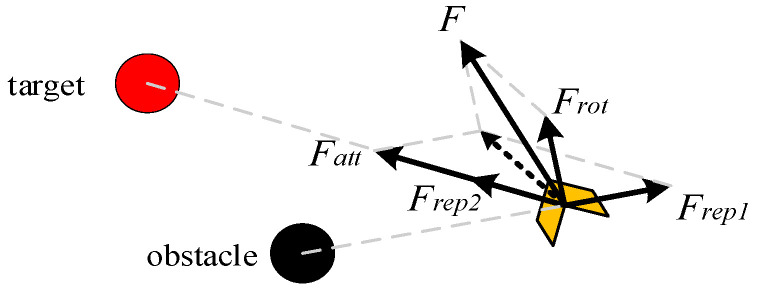
Force model of the new artificial potential field method.

The force model of the new artificial potential field method (NAPF) with an improved repulsion field is shown in Figure 5.

The repulsion potential field is improved as follows:

(1) $$\left\{\begin{array}{cc} k_{1}(\frac{1}{f\left(q-q_{o}\right)}-\frac{1}{\rho }{)}^{2}f^{n}(q-q_{g}), & f\left(q-q_{o}\right)\leq \rho \\ 0, & f\left(q-q_{o}\right)>\rho \end{array}\right.$$

where *n* is a constant greater than 0; *k*_1_ is the repulsion influence factor; *q_o_* is the obstacle position; *q* is the robot position; *q_g_* is the target position; *f*(*q − q_o_*) is the distance between the robot and the obstacle; and *p* is the obstacle influence distance.

The improved repulsion force is as follows:

(2) $$F_{rep}=-grad\left(U_{rep}\left(q\right)\right)=\left\{\begin{array}{cc} {F_{rep}}_{1}+F_{rep2}, & f\left(q-q_{o}\right)\leq \rho_{o}\\ 0, & f\left(q-q_{o}\right)>\rho_{o} \end{array}\right.$$

In the formula, *F_rep_*_1_ and *F_rep_*_2_ are as follows:

(3) $$F_{rep1}=2k_{r}\left(\frac{1}{f\left(q-q_{o}\right)}-\frac{1}{\rho }\right)\frac{f^{n}\left(q-q_{g}\right)}{f^{2}\left(q-q_{o}\right)}grad\left(f\left(q-q_{o}\right)\right)$$

(4) $$F_{rep2}=k_{r}\left(\frac{1}{f\left(q-q_{o}\right)}-\frac{1}{\rho }\right)\frac{f^{n-1}\left(q-q_{g}\right)}{f^{2}\left(q-q_{o}\right)}grad\left(f\left(q-q_{g}\right)\right)$$

Among them, the *F_rep_*_1_ direction is the direction in which the obstacle points to the robot; the *F_rep_*_2_ direction is the direction in which the robot points to the target point. By strengthening the attraction of the target point, the robot is encouraged to quickly pass through obstacles and avoid oscillation.

#### 3.1.2. Density Detection Extension Mechanism

**Figure 6:**
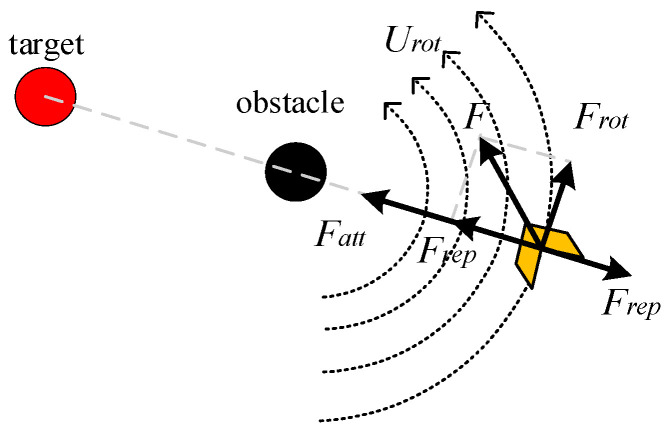
Action diagram of the rotating force.

The rotating potential field is defined as *U_rot_*, and the rotating force *F_rot_* generated by it is used to guide the robot to escape from the local minimum trap and solve the target unreachable problem, as shown in Figure 6.

The center of the rotating potential field, *U_rot_*, is in the center of the obstacle, and its direction is counterclockwise along the obstacle. The closer to the obstacle, the stronger the potential field. The rotating potential field can be expressed as:

(5) $$U_{rot}\left(q\right)=\left\{\begin{array}{cc} \frac{1}{2}k_{e}(\frac{1}{f\left(q-q_{o}\right)}-\frac{1}{\rho }{)}^{2} & f\left(q,q_{o}\right)\leq \rho \\ 0 & f\left(q,q_{o}\right)>\rho \end{array}\right.$$

where *k_e_* represents a positive scaling factor, *q_o_* represents the position of the obstacle, *f*(*q − q_o_*) represents the distance between the robot and the obstacle, and *p* represents the effective range of the obstacle potential field. The purpose of introducing the rotating potential field is to solve the problem of unreachable targets. Once the robot enters the obstacle region, the rotating force *F_rot_* continues to be generated, thus avoiding a situation where the robot has zero force. The calculation definition of the rotating force *F_rot_* is as follows:

(6) $$\left\{\begin{array}{cc} k_{e}\left(\frac{1}{f\left(q-q_{o}\right)}-\frac{1}{\rho }\right)\frac{1}{f^{2}\left(q-q_{o}\right)}grad\left(f\left(q-q_{o}\right)\right), & f\left(q-q_{o}\right)\leq \rho \\ 0, & f\left(q-q_{o}\right)>\rho \end{array}\right.$$

### 3.2. Internal Obstacle Avoidance Mechanism of Multiple Robots

When avoiding obstacles, multiple mobile robots must consider not only the collisions between all robots and static obstacles, but also the avoidance of internal collisions between robots. To solve the collision and conflict problem between multiple mobile robots, this paper combines the artificial potential field method with formation conversion to achieve conflict avoidance. First, a safe distance between the robots, *L*, is set, and then the follower robots are numbered and divided into left and right groups, centered on the leader. When any group of robots detects obstacles, they enter formation conversion mode. Owing to consistency control in the formation conversion mode, there are no intrateam collisions. Specific details are introduced in Section 5.

**Figure 7:**
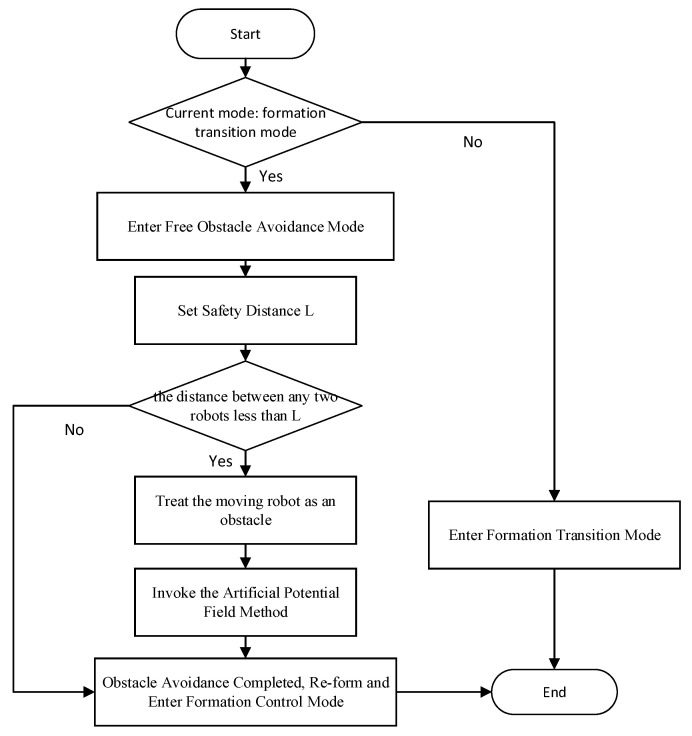
Flow chart of the obstacle avoidance principle.

If there is still a possibility of collision with obstacles after the formation change, robots will enter the free obstacle avoidance mode. In this mode, the robots leave formation control in time, and each robot avoids obstacles autonomously according to the improved artificial potential field method. If the distance between a robot and other robots is less than the safe distance *L* because of obstacle avoidance, the other robots are treated as dynamic obstacles to avoid intrateam conflict, a repulsion force field is given to it, and a corresponding repulsion force is generated. Because safety is the primary goal of conflict avoidance within the robot team, the repulsion field only needs to use the repulsion field of the original artificial potential field method. After all the mobile robots have successfully completed obstacle avoidance, they continue to reorganize the formation to ensure the formation remains. The pseudocode of the dynamic obstacle avoidance of mobile robots is shown in Algorithm 4.
**Algorithm 4** InterAvoidance (Num, Frep, RobotPose, L)1: for *i* = 1 to N do  2:    *l* = GetDistances(FollowerPose(*i*)) 3:    **if**
*l < L* && *l* ≠ *0*
**then** 4:   *Frep*
**=**
*Frep* + ComputeRepulsion(*RobotPose* (*Num*), *RobotPose*(*i*))  5:    **end if** 6: **end for** 7: **return**
*Frep*;

Where *Num* is the current robot number, *Frep* is the repulsion force obtained by the current robot, *RobotPose* stores the real-time position coordinates of all the robots, and *N* is the total number of robots. The other robots are traversed in turn, and the position distance *l* is calculated. If *l* is less than the safe distance and is not 0, the traversed robots are treated as obstacles, and the repulsion force is recalculated. When all the robots have completed the traverse, the final repulsion force is output. The repulsion force at this time considers both the obstacle and the other internal robots so that internal collisions can be avoided. The flow chart of the obstacle avoidance principle is shown in Figure 7.

## 4. Multirobot Formation and Formation Conversion Model

### 4.1. Multirobot Cooperative Control

This research adopts a distributed control structure of leader–follower formation and combines graph theory and a consistency algorithm to achieve formation control. The first-order continuous model of the system is as follows:

(7) $${\dot{x}}_{i}=u_{i},\left\{x_{i},u_{i}\in R^{n}\right\}$$

where *x_i_* represents the state quantity of the *i*-th robot, *u_i_* represents the input quantity of the *i*-th robot, and *n* represents the dimension of the state quantity. Ideally, the control input quantity is as follows:

(8) $$u_{i}=\sum_{j\in N_{i}}a_{ij}\left(x_{j}-x_{i}\right)$$

where *a_ij_* is the adjacency matrix element of the team and *N_i_* is the neighbor set of member *i*. Equations (7) and (8) are introduced into the global state vector $x={\left[x_{1},x_{2},\ldots ,x_{n}\right]}^{T}\in R^{n}$, and Equations (1)–(3) are combined to obtain the global dynamic relationship Equation (9):

(9) $${\dot{x}}_{i}=-Dx+Ax=-Lx$$

Equation (10) shows that the Laplace matrix *L* affects the closed-loop dynamic characteristics of the entire formation. If and only if there is a spanning tree in the topological graph *G* are the eigenvalues of the −L matrix located on the left side of the complex plane; thus, Equation (8) can achieve system consistency, and the final state value is as follows:

(10) $$c=\sum_{i=1}^{N}p_{i}x_{i}\left(0\right)$$

In Equation (7), the state quantity of the mobile robot is further discretized into:

(11) $$x_{i}\left(k+1\right)=x_{i}\left(k\right)+u_{i}\left(k\right)$$

The number of leaders is set as the last *N* of the robot number, and its control algorithm is as follows:

(12) $$u_{N}\left(k\right)=m+kD\left(k\right)+\sum_{i\in N_{i}}a_{Ni}r_{Ni}\left(k\right)$$

where *m* and *n* are constants, *r_Ni_*(*k*) represents the relative position of the leader and other robots at time *k*, and *D*(*k*) represents the distance from the leader to the target point at time *k*.

The followers need to follow the leader to move and maintain a certain formation, and its control algorithm is as follows:

(13) $$u_{i}\left(k\right)=\varepsilon \sum_{j\in N_{i}}a_{ij}\left(x_{j}\left(k\right)-x_{i}\left(k\right)-r_{ij}\left(k\right)\right)$$

where ε is a constant greater than 0, and *r_ij_*(*k*) represents the relative position relationship between robot *i* and robot *j* at time *k*.

### 4.2. Formation Conversion Mechanism

**Figure 8:**
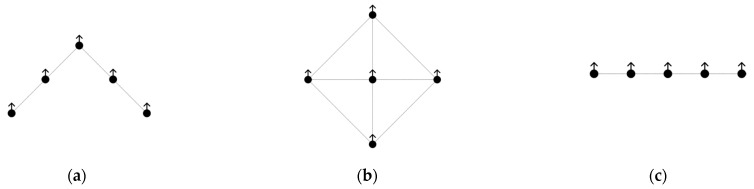
Formation examples. (**a**) Geese formation (**b**) Diamond formation (**c**) Line formation.

Multiple mobile robot formations play important roles in various scenarios. These include search, rescue, and military confrontations. By moving in formation, the robot team can quickly respond to task requirements, improve work efficiency, reduce energy consumption, and extend the service life of the robot. The commonly used team formations include wild geese, diamonds, lines, and other formations, as shown in Figure 8.

**Figure 9:**
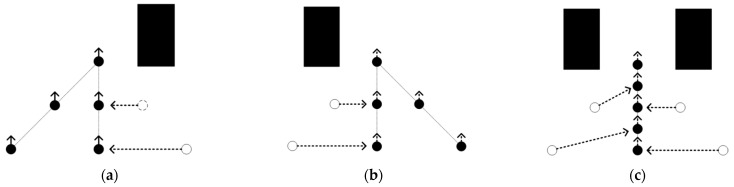
Formation conversion mechanism. (**a**) Transformation strategy when there is an obstacle on the right side of the line. (**b**) Transformation strategy when there is an obstacle on the left side of the line. (**c**) Transformation strategy when there are obstacles on both sides of the line.

The wild goose formation allows the robots to be reasonably distributed in a limited space, optimize path selection, and change the formation in a timely manner when crossing a narrow zone to reduce the risk of blockage and collision. In terms of search and rescue, the wild goose formation can also significantly enhance team search efficiency. In this study, the wild goose formation was used as the basic formation. To ensure that the team can avoid obstacles in an orderly manner when encountering obstacles, three formation conversion methods are proposed to correspond to three obstacle encounter situations. The followers on the left and right sides of the leader are numbered. When the followers on the right side detect obstacles, the followers on the right side move closer to the middle, and the followers on the left side do the same to ensure that the team avoids obstacles efficiently. As shown in Figure 9a,b, when both the left and right robots detect obstacles, the formation becomes a line formation, as shown in Figure 9c.

### 4.3. Formation Steering Mechanism

**Figure 10:**
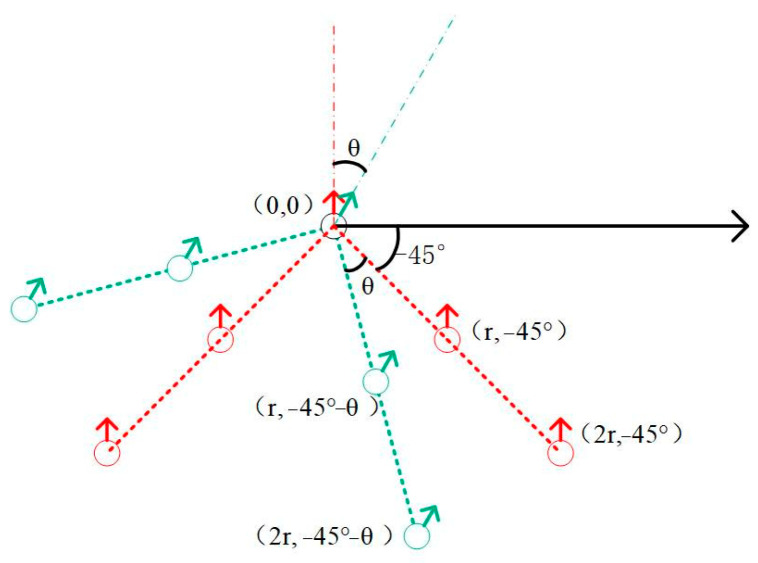
Coordinate conversion after formation steering.

Since formation maintenance is realized according to the relative position relationship between robots, this paper adopts the Decarr coordinate system to represent the coordinates of the robots. When the leader needs to make a turn, the whole formation should also follow the leader to make a turn, so the relative position coordinates should also always change. To ensure that the formation turns simultaneously with the leader, this paper proposes a position relationship based on polar coordinate conversion, as shown in Figure 10. The relative position relationship of the initial red wild goose formation in the figure is represented in polar coordinates as follows: leader (0,0), right follower $\left(r,-45^{\circ }\right)$, $\left(2r,-45^{\circ }\right)$, left follower $\left(r,-135^{\circ }\right)$, and $\left(2r,-135^{\circ }\right)$. When the leader turns at time *k*, a steering angle *θ* is generated, and the formation also needs to turn. The relative position relationship is expressed in polar coordinates as follows: the leader remains unchanged (0,0), the right follower becomes $\left(r,-45^{\circ }-\theta \right)$, $\left(2r,-45^{\circ }-\theta \right)$, and the left follower becomes $\left(r,-135^{\circ }-\theta \right)$, $\left(2r,-135^{\circ }-\theta \right)$. After the new position relationship is changed, Equation (14) is used to change the polar coordinate position into a rectangular coordinate position relationship. The rectangular coordinate position relationship is then brought into the relative position *r_Ni_*(*k*) at time *k* in Equation (12) to update the formation to complete the turn.

(14) $$\left\{\begin{array}{c} x=r*\cos\theta \\ y=r*\sin\theta \end{array}\right.$$

## 5. Simulation and Results

To verify the proposed algorithm, this paper compares and analyzes the rope path optimization algorithm under various maps. The improved artificial potential field methods are compared and analyzed under the multi-obstacle map. Finally, multirobot path planning and collaborative formations are verified and analyzed via a comprehensive map. The size of all the global maps is 100 px × 100 px, and the size of the artificial potential field method experiment scene is 35 px × 35 px. Among them, 1 px represents 1 m in the real world. All the simulations are performed on a computer equipped with an Intel(R) Core(TM) i7-12700H 2.30 GHz CPU and 16 GB of RAM. The simulation platform for all the experiments is MATLAB R2022a.

### 5.1. Global Path Planning

The global path planning experiment parameters are set as shown in Table 1. Among them, *d_stepsize_* is the random tree expansion step, *d_step_* is the movement distance of the path point when the rope contracts, *r* is the end point detection radius, and *N* is the maximum sampling number.

**Figure 11:**
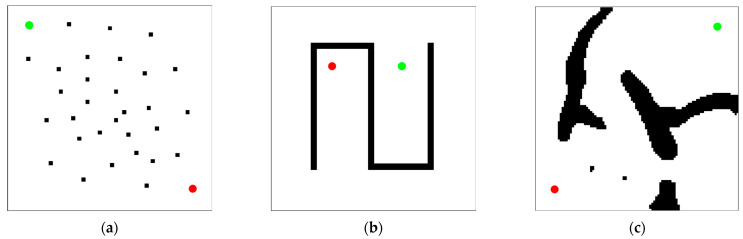
Three experimental environments. (**a**) Random scatter map (**b**) Maze map (**c**) Map simulating a real-world scenario.

This paper tests the performance of the DDRRT algorithm in three two-dimensional environments. The simple environment shown in Figure 11a is used to test whether the performance of the new algorithm is significantly reduced due to additional calculations. The U-shaped maze environment shown in Figure 11b is used to test the performance of the algorithm in a complex environment. The simulation environment shown in Figure 11c is used to evaluate the actual performance of the algorithm. In the map, the red dots are the starting points, and the green dots are the target points. The entire exploration process is represented by green lines, and the generated path is represented by blue lines. Each experiment is repeated 100 times, and algorithm performance is evaluated based on the average algorithm running time, minimum time, maximum time, number of tree nodes, and time standard deviation.

**Figure 12:**
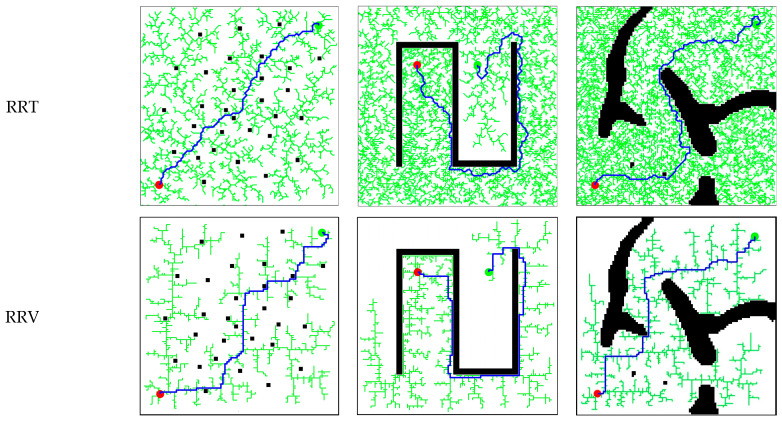

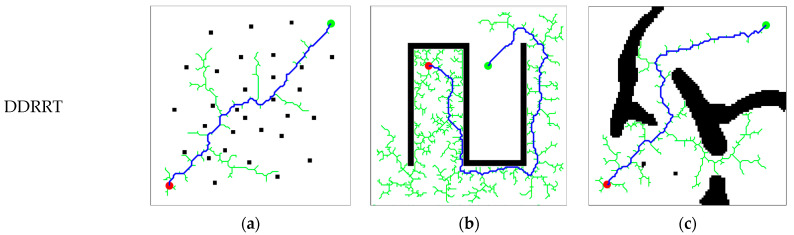
Comparative experiment of global path planning algorithms. (**a**) Simulation experiment under the scatter plot map (**b**) Simulation experiment under the maze map (**c**) Simulation experiment under the simulation map.

Figure 12 illustrates the operations of DDRRT, RRT, and RRV [17] (rapidly exploring random vines) in three different map environments. Regardless of the environment, DDRRT generates significantly fewer tree nodes than both RRT and RRV. The randomly generated tree is more concise than those of RRT and RRV, intuitively demonstrating that DDRRT has a higher utilization rate of tree nodes.

Table 2 presents the operational data of the algorithms in the three map environments. The planning time of DDRRT in different environments is less than that of both the RRT and RRV algorithms, indicating that DDRRT has higher planning efficiency. The time standard deviation of DDRRT is smaller than that of RRT and RRV, suggesting that DDRRT is more stable in the algorithm dataset. Additionally, the average number of tree nodes in DDRRT is significantly smaller than that of the original RRT and RRV algorithms, further indicating its higher node utilization rate.

Based on the above analysis, in different environments, the DDRRT algorithm outperforms both the original RRT and RRV algorithms. The reason lies in DDRRT’s addition of a target-oriented expansion mechanism and continuous detection of vertex density to avoid redundant exploration. This improves vertex utilization and enhances the overall performance of the algorithm.

### 5.2. Global Path Optimization

**Figure 13:**
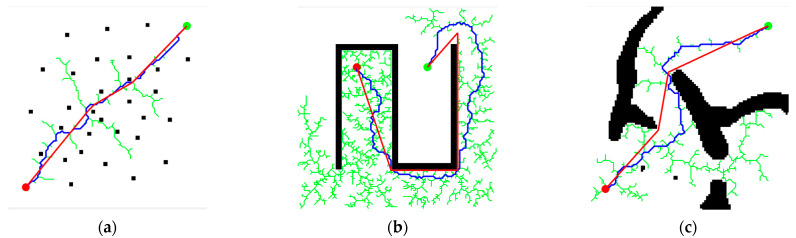
Path optimization comparison. (**a**) Path optimization under the scatter plot map (**b**) Path optimization under the maze map (**c**) Path optimization under the simulation map.

Although DDRRT has great advantages in terms of planning speed and environmental adaptability, there is still room for improvement in the quality of the planned path. Therefore, to solve this problem, this paper proposes a rope path optimization strategy to optimize the path. This paper uses three different maps to verify the feasibility of the rope path optimization method and ensure that it can provide high-quality paths for leaders in different terrains. Each experiment is repeated 100 times, and the quality of the path is evaluated based on path length and smoothness. Figure 13 shows the simulation results on three maps. The blue line represents the initial path obtained by the RRT based on its own expansion tree, and the red line represents the path optimized by the rope path.

Obviously, the blue paths under the three maps are too tortuous and contain many redundant path points, making it difficult to provide an effective global path for the leader. The red path has almost no redundant path points, and each point can be used as a local destination to provide better guidance for the leader.

Table 3 compares and analyzes the quality of the paths generated in the experiment. On the three different maps, the optimized path has significant improvements in path length and smoothness, and is close to the optimal path. This finding shows that rope path optimization can effectively improve the RRT initial path quality.

### 5.3. Local Obstacle Avoidance Optimization Strategy

To verify the superiority of the improved new artificial potential field (NAPF) in the field of local path planning, this paper compares NAPF with the traditional artificial potential field method in rectangular obstacle channels. The map size is 35 px × 35 px. The experimental parameter settings are shown in Table 4. Among them, *kg* is the attraction gain coefficient, *kr* is the repulsion gain coefficient, *ke* is the rotating force gain coefficient, *ρ* is the radius of the obstacle repulsion field, and *v_max_* is the maximum driving speed.

**Figure 14:**
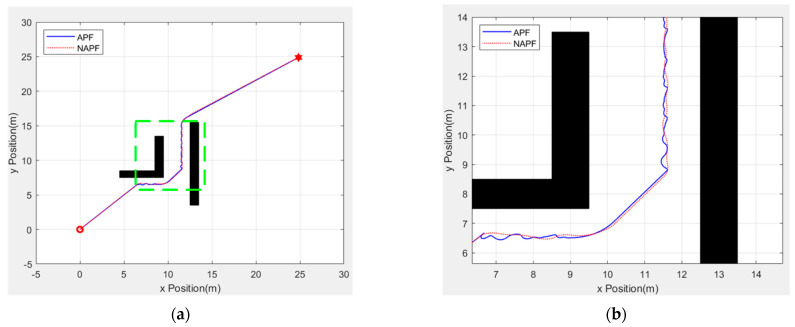
Comparison experiment with a rectangular obstacle map. (**a**) Panoramic view (**b**) Zoomed-in view of the green box in image (**a**).

Figure 14 shows the obstacle avoidance paths planned by two artificial potential field algorithms in a channel environment with rectangular obstacles. Among them, the starting point and ending point remain unchanged. Figure 14a shows that the two obstacle avoidance algorithms have similar path trajectories in this scenario, and both can avoid obstacles and reach the destination. Figure 14b is an enlarged image of the green rectangle in Figure 14a. When the traditional artificial potential field method encounters rectangular obstacles, the path is tortuous because the repulsion force and the attraction force are difficult to balance. The path of the new artificial potential field method is smoother. The reason is that the repulsion field has been improved to greatly increase the attraction force, allowing for obstacles to quickly be avoided when encountered.

Table 5 further shows the operating data of the two algorithms. The data show that using the new artificial potential field method reduces the driving time by 23%, reduces the path length by 2%, and improves path smoothness by 81%. These data further prove that the new artificial potential field method has obvious advantages in performance.

### 5.4. Robot Cooperative Formation

Based on the global path obtained by the leader, this paper combines the consistent control algorithm and the artificial potential field method to realize multirobot formation driving. To ensure that the robot team can avoid obstacles while maintaining formation stability when encountering obstacles, this paper proposes a formation conversion mechanism based on polar coordinate conversion and combines it with a new artificial potential field method to realize multirobot formation driving. To verify the effectiveness of the proposed strategy, it is compared with the traditional strategy without a formation conversion mechanism.

**Figure 15:**
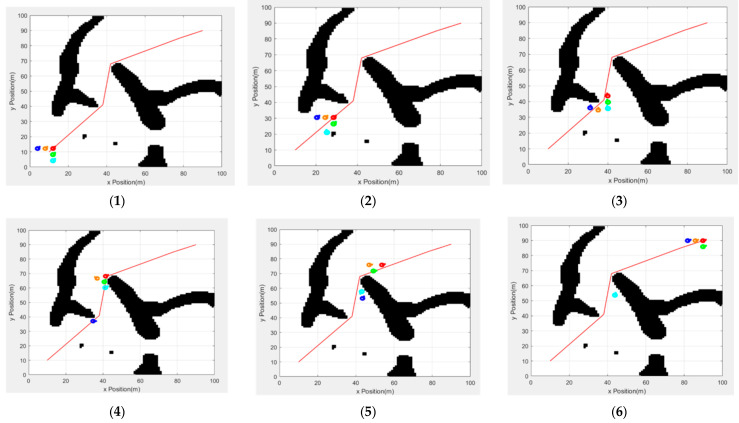
Traditional artificial potential field methods and driving without formation changes. The images from (**1**–**6**) are screenshots of the formation movement in chronological order.

Figure 15 shows the formation driving process of the traditional artificial potential field method without a formation conversion mechanism. Figure 15(1) shows the beginning of the experiment. The team’s formation is based on the wild goose formation, and the leader runs in turn with the path point of the global path as the stage target. Figure 15(2) shows the autonomous obstacle avoidance process of the light blue follower robot when the team encounters obstacles for the first time. Figure 15(3) shows the process of the leader turning and changing the target when reaching the stage target point. At this time, owing to the lack of a formation conversion mechanism, the formation has gradually collapsed. Figure 15(4) shows the situation in which the leader reaches the next target point. At this time, the dark blue follower separates from the team due to obstacles; Figure 15(5) shows the leader’s driving process toward the end point. The dark blue and light blue followers separate from the team again due to obstacles; the formation has completely collapsed. Figure 15(6) shows that the leader has reached the end point. However, the light blue follower has not yet returned to the team, resulting in mission failure.

**Figure 16:**
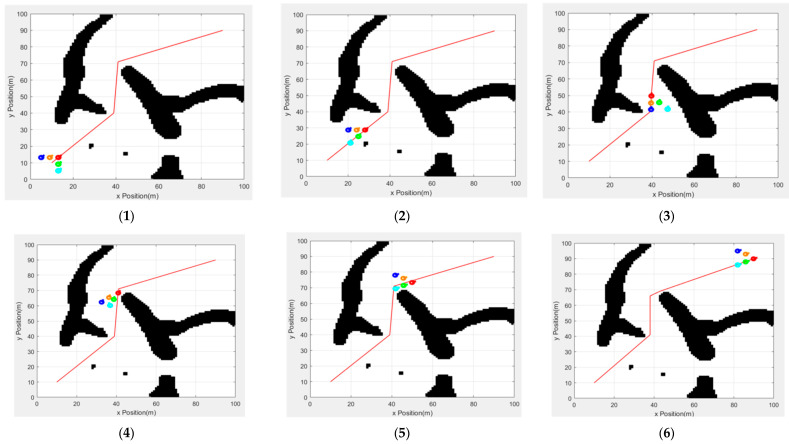
The new artificial potential field method is combined with formation changes. The images from (**1**–**6**) are screenshots of the formation movement in chronological order.

Figure 16 shows the formation driving process combined with the formation conversion mechanism and the new artificial potential field method. In Figure 16(1), at the beginning of the experiment, the team is based on the wild goose formation, and the leader runs in turn with the path point of the global path as the stage target. In Figure 16(2), the team encounters obstacles for the first time. The right-side followers find the obstacle and change formation to move closer to the middle. In Figure 16(3), when the stage target point is reached, the leader switches the target. At this time, the left-side followers detect that the obstacle is moving closer to the middle, and the right-side followers spread out. In Figure 16(4), the leader reaches the next target point and is ready to turn. At this time, the followers on the right side detect that the obstacle is moving closer to the middle, and the formation follows the leader to turn. In Figure 16(5), the leader is driving toward the end point, and the formation follows the leader to complete the turn. Finally, in Figure 16(6), the leader reaches the end point. All the robots successfully reach the end point, maintain a certain formation during driving, and successfully complete the task.

**Figure 17:**
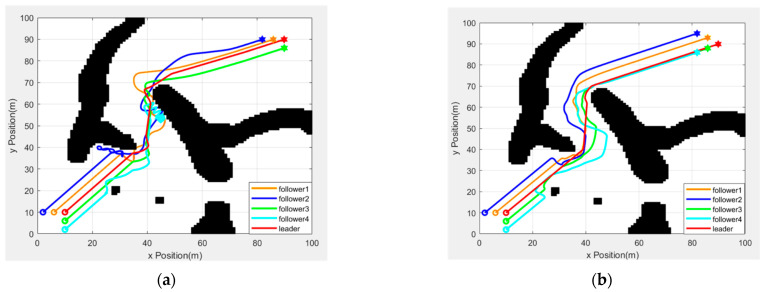
Comparison of robot driving trajectories. (**a**) Formation movement trajectory under the original algorithm (**b**) Formation movement trajectory after the improved algorithm.

Figure 17 shows the formation trajectory diagram of the robots in the two experiments. In Figure 17a, the trajectory without the formation conversion mechanism is shown. Figure 17b shows the trajectory after the formation conversion mechanism is introduced. After introducing the formation conversion mechanism, the robot team can avoid obstacles via conversion when encountering obstacles and avoid the loss of followers. In contrast, the trajectory without the formation conversion mechanism is more chaotic. This means that the overall formation is not working well, and that some followers go missing. This further proves the importance of the formation conversion mechanism.

## 6. Summary

With respect to the leader–follower formation control method, this paper proposes a sample-based global path planning algorithm, DDRRT, to provide high-quality global paths for the leader. The algorithm restricts the selection and expansion of tree vertices through density detection to improve the utilization rate of each vertex as much as possible. To address the low quality of the path generated by the sampling algorithm, a rope contraction path optimization strategy is proposed. The initial path is simulated as a rope and stretches at both ends to reduce the path length and improve the path quality. This paper improves the potential field function of the artificial potential field method and introduces a rotating potential field to solve the local obstacle avoidance problem of multiple mobile robots. Finally, to solve the problems of slow obstacle avoidance and inability to turn into the consistency control algorithm, an obstacle avoidance mechanism based on formation conversion and a formation turning strategy based on polar coordinate conversion are proposed. Experimental simulations show that the proposed algorithm runs stably and can realize multirobot cooperative formation operations. Although DDRRT performs well in simulation experiments, it still has some limitations in practical applications. First, DDRRT’s computational complexity is relatively high, especially in high-dimensional state spaces or large-scale robot formations, which may limit its real-time performance. Second, DDRRT is primarily designed for static environments, and in dynamic environments (e.g., with moving obstacles or uncertain conditions), it needs to be combined with prediction models or methods such as the Dynamic Window Approach (DWA) to improve adaptability. Additionally, DDRRT relies heavily on sensor accuracy and environmental modeling, and its performance may degrade in real-world applications due to sensor noise or environmental modeling errors. Future research will focus on reducing DDRRT’s computational complexity, enhancing its real-time performance in dynamic environments, and exploring its potential in more complex scenarios.

## Figures and Tables

**Table 1:** Experimental parameter settings.

Parameter	*d_stepsize_* (px)	*d_step_* (px)	*r* (px)	*N*
Value	1	0.2	1	3000

**Table 2:** Experimental simulation data.

Map Environment	Algorithm	Average Time	Minimum Time	Maximum Time	Time Standard Deviation	Average Tree Node Number
Simple simulation environment	RRT	4.27	1.36	8.98	5.81	4271
	RRV	0.82	0.67	1.24	2.57	582
	DDRRT	0.23	0.17	0.27	1.39	327
U-shaped maze environment	RRT	6.74	1.79	12.43	13.97	6742
	RRV	5.69	4.52	6.65	3.27	1520
	DDRRT	0.38	0.22	0.74	2.75	462
Real simulation environment	RRT	7.42	2.38	17.15	19.62	9524
	RRV	2.24	0.97	3.82	7.42	3284
	DDRRT	0.91	0.65	1.27	2.35	1573

**Table 3:** Path quality comparison data table.

Map	Scatter Map		Concave Maze Map		Comprehensive Map	
Path	Length	Smoothness	Length	Smoothness	Length	Smoothness
Initial path	129	56.55	234	94.25	164	83.25
Optimized path	113	0.23	172	5.22	124	1.90
Optimal path	113	0.23	171	5.20	121	1.51

**Table 4:** Experimental parameter settings.

Parameter	kg	kr	ke	*ρ*	Vmax (m/min*)*
Value	5	15	10	1.5	2.5

**Table 5:** Experimental data under the channel obstacle map.

Algorithm	Driving Time (min)	Path Length (m)	Path Smoothness	Extreme Point
APF	19.85	39.34	63.49	0
NAPF	15.35	38.51	12.54	0

## Data Availability

The data used to support the findings of the study can be obtained from the corresponding author upon request.
